# Office Work Exposures and Adult-Onset Asthma

**DOI:** 10.1289/ehp.9875

**Published:** 2007-02-26

**Authors:** Maritta S. Jaakkola, Jouni J.K. Jaakkola

**Affiliations:** 1 Institute of Occupational and Environmental Medicine, University of Birmingham, Birmingham, United Kingdom; 2 Finnish Institute of Occupational Health, Helsinki, Finland; 3 Environmental Epidemiology Unit, Department of Public Health, University of Helsinki, Helsinki, Finland

**Keywords:** asthma, carbonless copy paper, case–control study, paper dust, photocopiers, population-based

## Abstract

**Background:**

Office exposures have been linked to symptoms of sick building syndrome, but their relation to the development of asthma has not been studied previously. These exposures have increasing importance because an increasing proportion of the workforce is working in office environments.

**Objectives:**

The aim of this study was to assess the relations of exposure to carbonless copy paper (CCP), paper dust, and fumes from photocopiers and printers to adult-onset asthma.

**Methods:**

We conducted a population-based incident case–control study of adults 21–63 years of age living in the Pirkanmaa District in South Finland. All new clinically diagnosed cases (*n* = 521) of asthma were recruited during a 3-year study period. A random sample of the source population formed the controls (*n* = 1,016). This part focused on 133 cases and 316 controls who were office workers according to their current occupation classified by the 1988 International Standard Classification of Occupations. All participants answered a questionnaire on health, smoking, occupation, and exposures at work and home. Subjects with previous asthma were excluded.

**Results:**

Exposures to paper dust [adjusted odds ratio (OR) = 1.97; 95% confidence interval (CI), 1.25–3.10] and CCP (OR = 1.66; 95% CI, 1.03–2.66) were related to significantly increased risk of adult-onset asthma. An exposure–response relation was observed between exposure to paper dust and risk of asthma.

**Conclusions:**

This study provides new evidence that exposures to paper dust and CCP in office work are related to increased risk of adult-onset asthma. Reduction of these exposures could prevent asthma in office workers. Clinicians seeing asthma patients should be aware of this link to office exposures.

In the modern world, an increasing proportion of the workforce is working indoors in offices. As this trend continues, the exposures in office environments are likely to influence the health, well-being, and productivity of more and more employees. Although the physical office environment has traditionally been considered safe, a few previous studies have linked some office exposures to symptoms of sick building syndrome, such as eye, nose, throat, and skin symptoms, cough, and fatigue. The office exposures that have been linked to these symptoms include carbonless copy paper (CCP) (Jaakkola and Jaakkola 1999; Morgan and Camp 1986; Skov et al. 1989) and fumes from photocopiers and printers (FPP) (Fisk et al. 1993; Jaakkola and Jaakkola 1999; Skov et al. 1989; Stenberg et al. 1993). One previous study, the Helsinki Office Environment Study, linked exposure to carbonless copy paper also to occurrence of lower respiratory symptoms, such as wheezing, cough, phlegm production, and chronic bronchitis (Jaakkola and Jaakkola 1999). According to a systematic Medline search (http://gateway.uk.ovid.com/gwl.ovidweb), no previous study had addressed the effects of these office exposures on incident asthma, although asthma is a rather common chronic disease in working-age adults. It is not uncommon in clinical settings that patients themselves link their asthmatic symptoms to office exposures, such as paper dust and CCP.

The objective of our study was to assess the relations between exposures to CCP, paper dust, and FPP and adult-onset asthma in a population of office workers in Finland.

## Methods

### Study design

This study was a population-based incident case–control study of adult-onset asthma. The source population consisted of adults 21–63 years of age living in a geographically defined administrative area (Pirkanmaa) in South Finland, with a population of 440,913 in 1997.

The study was approved by the ethics committees of the Finnish Institute of Occupational Health and the Tampere University Hospital. All participants gave informed consent before participating in the study.

### Study population

#### Cases

From September 1997 to March 2000, we systematically recruited all new cases of adult asthma diagnosed in the study area by recruitment through all health care facilities that diagnose asthma, including the Department of Respiratory Medicine at the Tampere University Hospital, and all health care centers and private practicing physicians. In addition, the National Social Insurance Institution of Finland, which grants the reimbursement right for asthma medications, invited new asthmatics who had not been identified by the original thorough recruitment system but were identified by a computer search of their files. The diagnostic criteria of asthma included occurrence of at least one asthma-like symptom and demonstration of reversible airways obstruction in lung function investigations (Appendix 1) (Jaakkola JJK et al. 2002, 2003; Jaakkola MS et al. 2002, 2003, 2006; National Asthma Program in Finland 1994; Piipari et al. 2004).

At the Tampere University Hospital, all potential cases referred for suspicion of asthma were recruited at their first visit, and the diagnosis was then verified in clinical examinations. Only those cases for whom the criteria of asthma were fulfilled were included in the final study population. At the other health care facilities, cases were recruited immediately when their asthma diagnosis was confirmed. The National Social Insurance Institution invited cases 0.5–2 years after their diagnosis was established (according to the requirements for reimbursement rights). For these patients, the date and criteria of the asthma diagnosis were confirmed from their medical records to ensure that their diagnosis fulfilled the criteria of our study protocol followed by the other health care facilities.

Eligible subjects were invited to participate in the study, and informed consent was requested by their physician or through a letter sent by the National Social Insurance Institution at the time of recruitment into the study. The medical records of all cases were checked, and only those with no previously diagnosed asthma or long-term use of any asthma medication were included in the study. A total of 521 cases (response rate 86%) participated in the study. Of these, 133 cases were professionals, clerks, or administrative personnel according to their current occupation as classified by the 1988 International Standard Classification of Occupations (ISCO-88) (International Labor Organization 1988), and these form the case population for this study.

#### Controls

Controls were randomly drawn from the source population (21- to 63-year-old residents of Pirkanmaa District) using the national population registry (Population Register Centre; http://www.vaestorek-isterikeskus.fi/vrk/home.nsf/pages/index_eng), which has full coverage of the population. Recruitment took place by a letter at 6-month intervals throughout the study period. Before sending the letter of invitation, we checked from the population registry whether the person still lived in the study area and excluded those who had moved (or died) from the target population. Of the original sample of 1,500 potential controls randomly selected in 1997, 1,270 were included as controls in the target population. Altogether, 1,016 controls participated in the study (response rate 80%). After exclusion of 76 persons reporting previous/current asthma (7.5%), six persons > 63 years of age (0.6%), and two who returned incomplete questionnaires (0.2%), the number of controls was 932. Of these, 316 were professionals, clerks, or administrative personnel according to their current occupation as classified by the ISCO-88.

### Measurement methods

#### Questionnaire

At the time of recruitment, the study subjects answered a self-administered questionnaire modified from the Helsinki Office Environment Study questionnaire (Jaakkola and Jaakkola 1999; Jaakkola and Miettinen 1995) for use in a general population (Jaakkola JJK et al. 2002, 2003; Jaakkola MS et al. 2002, 2003, 2006; Piipari et al. 2004). The questionnaire included six sections: *a*) personal characteristics; *b*) health information, including respiratory symptoms and previous respiratory and allergic diseases; *c*) active smoking and environmental tobacco smoke (ETS) exposure; *d*) occupation and exposures in the work environment; *e*) exposures in the home environment; and *f*) dietary questions. The fourth section requested information on current occupation and previous occupations throughout the working history. It also included additional questions on indoor environment at work, including exposures to paper dust, CCP, FPP, and occurrence of dampness and mold problems. The question on exposure to paper dust, CCP, and FPP asked the study subjects to assess their average exposure in hours per week in their current job. If their asthmatic respiratory symptoms had started in another job, the same information was asked for this job. In the analysis, we used exposures in the current job or, for those whose symptoms had started in any previous job, their exposures in that job.

#### Lung function measurements

The same diagnostic protocol was applied for all patients with suspicion of asthma (Appendix 1) (Jaakkola JJK et al. 2002, 2003; Jaakkola MS et al. 2002, 2003, 2006; National Asthma Program in Finland 1994; Piipari et al. 2004). The baseline spirometry and bronchodilation test were recorded with a pneumotachograph spirometer connected to a computer and using a disposable flow transducer (Medikro 905; Medikro, Kuopio, Finland) according to the standards of the American Thoracic Society (1995). We judged presence of obstruction using reference values derived from the Finnish population (Viljanen et al. 1982). All patients performed peak expiratory flow (PEF) follow-up with measurements twice a day for at least 2 weeks using a mini-Wright meter (Clement Clarke International Ltd., Essex, UK). During the second week, measurements were taken before and 15 min after short-acting bronchodilating medication. A 2-week oral steroid treatment with 20 mg pred-nisolon was carried out for those with a strong suspicion of asthma, if the other diagnostic tests were negative. The patient performed 2 more weeks of PEF follow-up during this treatment as well as a spirometry at the end of the treatment period to judge the response to the medication.

### Statistical methods

Statistical analyses were performed using SAS statistical package (SAS Institute Inc., Cary, NC, USA). We used exposure odds ratio (OR) to quantify the relation between exposures to paper dust, CCP, or FPP and incident asthma, estimated in logistic regression analysis adjusting for sex, age, education (as an indicator of socioeconomic status), personal smoking status (never, former, current), exposure to ETS (at work and/or home vs. none), and exposure to indoor mold problems (at work and/or home vs. none). We investigated three types of models with respect to exposures: *a*) First, the models included each exposure of interest (paper dust, CCP, or FPP) as a dichotomous variable using the cut-off point of 1 hr/week (≥ 1 hr/week = exposed; < 1 hr/week = unexposed; the latter was the reference category), giving as estimate for any exposure; *b*) second, the models included each exposure of interest categorized into three groups: high exposure (≥ 30 hr/week for paper dust; ≥ 15 hr/week for CCP; and ≥ 5 hr/week for FPP), low exposure (1 to < 30, 1 to < 15, and 1 to < 5 hr/week, respectively), and no exposure (< 1 hr/week, the reference category), giving estimates for exposure response while not assuming a linear relation; *c*) third, the models included each exposure of interest as a continuous variable in hours per week, giving an estimate of exposure–response relation assuming a linear relation between exposure and log odds of asthma. Finally, a model including any exposure to paper dust, CCP, and/or FPP (≥ 1 hr/week) was fitted.

## Results

### Characteristics of the study population

There were a higher proportion of women, somewhat more of those with lower education, and more current smokers among cases than controls (Table 1). In multivariate analyses, we adjusted for all of these covariates.

### Distribution of office exposures and their relations to incident asthma

The distributions of exposures among cases and controls are presented in Table 2. The table also shows crude and adjusted ORs of incident asthma in adults in relation to office exposures. The risk of asthma was significantly increased in relation to any paper dust exposure [OR = 1.97; 95% confidence interval (CI), 1.25–3.10]. We observed an exposure-dependent increase in the risk of asthma with increasing exposure to paper dust (OR = 1.20 per 10 hr/week; 95% CI, 1.06–1.37). Any exposure to CCP was also significantly related to the risk of incident asthma (OR = 1.66; 95% CI, 1.03–2.66). Including CCP exposure as a continuous variable suggested a small increase in the risk per 10 hr CCP exposure/week (OR = 1.08; 95% CI, 0.92–1.27), but this did not reach statistical significance. Studying categorical exposure variables (high, low, no) showed an exposure–response relation with paper dust exposure, but no obvious exposure–response relation with CCP exposure. Including FPP exposure as a continuous exposure variable in the model suggested a small, almost significant increase in the risk of asthma (OR = 1.12 per 10 hr/week; 95% CI, 0.95–1.31), but no obvious relation to asthma was seen in the other models.

Exposure to any of the three exposures at office work (CCP, paper dust, FPP) was related to a significantly increased risk of asthma (OR = 1.90; 95% CI, 1.05–3.44) (Table 2).

## Discussion

We conducted a large population-based study of incident asthma in Finland (the Finnish Environment and Asthma Study) to identify occupational and environmental exposures that are of importance for adult-onset asthma. With an incidence rate of asthma of 0.9 cases per 1,000 person-years observed in our population, the present study corresponded to a follow-up of approximately 100,000 adults for 5.8 years (the denominator was approximately 581,000 person-years) (Jaakkola MS et al. 2006). About one third of the population was professionals, clerks, or administrative staff, meaning that a high proportion of the work-force worked in an office environment.

The study provided new evidence that exposures to paper dust and CCP are related to a significantly increased risk of incident asthma. An exposure–response relation was observed between increasing exposure to paper dust and risk of asthma. This could suggest that the mechanism underlying the effect of paper dust on asthma is irritant in nature. Studies in paper industries with high paper dust levels have suggested that paper dust can be highly irritative, and such exposure has been linked to mucosal irritation, nasal symptoms, cough, wheezing, and exercise-induced asthma (Hellgren et al. 2001; Järvholm et al. 1988; Kraus et al. 2002; Torén et al. 1994).

Any exposure to CCP showed a significant relation with incident asthma, but no obvious increase in risk with increasing CCP exposure was detected. This could suggest that the mechanism underlying the effect of CCP is sensitization, which can take place even in small exposure levels and is not directly related to the magnitude of exposure. CCP contains solvents and color-forming chemicals, and these have been suggested to be potential sensitizers (Jäppinen and Kanerva 2000; LaMarte et al. 1988; Marks et al. 1984; Mølhave and Grunnett 1981; Norbäck et al. 1988; Shehade et al. 1987). For example, CCP chemicals that have been identified as capable of inducing allergic reactions in humans include para-toluene sulfinate of Michler’s hydrol, crystal violet lactone, alkyphenol novolac resin, and diethylenetriamine (Kanerva et al. 1993; LaMarte et al. 1988; Marks et al. 1984; Shehade et al. 1987). This discussion on potential mechanisms is naturally speculative and in the future, studies with immunologic and histologic investigations should be conducted to address the mechanisms.

FPP did not show any obvious association with incident asthma, although the continuous exposure showed a slightly increased risk per 10 hr exposure/week. This question needs to be investigated further in the future, for example, by studies with more detailed characterization of the magnitude of exposure of individuals.

### Validity issues

In this population-based case–control study, we were able to recruit a high proportion of all new cases of asthma in a geographically defined area by a thorough recruitment in the health care system and with the help of the National Social Insurance Institution, which provides nationally reimbursement of asthma medications. Patients were recruited at all health care facilities diagnosing asthma in the study area. The response rate among control subjects was also relatively high (80%). Thus, any major selection bias is unlikely in this study.

The population-based design of this study guards against potential reporting bias of exposures, which could be a problem in studies selecting their population from office buildings with known problems. Our exposure assessment was based on self-report, and thus can be subject to some misclassification. However, the fact that significant relation with incident asthma was observed for some office work exposures (paper dust and CCP) but not for others (FPP) speaks against any significant reporting bias. Measurements of indoor chemicals could have supported our exposure assessment; on the other hand, the exposures of interest consist of mixtures of agents, and currently it is not well understood which chemicals are the most relevant for health effects. Indeed, exposure to a mixture may be more relevant for the health effects than exposure to any individual agent. Thus our exposure assessment approach based on reporting of exposure to the entire mixture has its strengths. A potential limitation of the study is that if exposures had been avoided because of symptoms experienced, the effect estimates of our study would underestimate the true effects. To reduce this problem, in the analyses we used information on exposures in the job where the person started to experience asthma symptoms for the first time.

Defining asthma on the basis of objective findings in extensive lung function measurements performed in accordance with the national guidelines (National Asthma Program in Finland 1994) reduces information bias concerning the outcome; the knowledge of exposures of the patient did not affect the diagnosis of asthma. Such knowledge might cause bias if the outcome assessment relied purely on doctor-diagnosed asthma or symptom reports. Because of the national health care and reimbursement system of asthma medications in Finland, we can be quite confident that we were able to identify and exclude those with previous asthma or long-term use of asthma medications, assuring us that our asthma cases were new. Applying the rather strict Finnish criteria for significant reversibility of airways obstruction helped us exclude those who had chronic obstructive pulmonary disease rather than asthma.

We adjusted the relations between office exposures and incident asthma for a number of confounders in logistic regression analysis to eliminate these factors, such as educational level and smoking, as potential explanations for our results.

### Synthesis with previous knowledge

Our study is the first one to address the role of office exposures, such as paper dust, CCP, and FPP, for development of adult-onset asthma in office workers. Our finding of increased risk of asthma in relation to paper dust exposure in office environment is consistent with previous studies conducted in other type of workforces. A cross-sectional study from Croatia found significantly higher prevalences of chronic cough (36.6%) and dyspnea (18.8%) among paper recycling workers compared with unexposed workers (18.4% and 4.6%, respectively) (Zuskin et al. 1998). Four percent of paper recycling workers had occupational asthma. A case–control study from Sweden found increased risk of reported asthma (OR = 2.1; 95% CI, 1.4–3.2) in relation to paper dust exposure in a population including all types of workforces in the city of Gothenburg (Torén et al. 1999). These previous studies did not investigate potential exposure–response relations. Our study showed an increase in the risk of asthma with increasing paper dust exposure.

Our finding of increased risk of incident asthma in relation to CCP exposure in office workers is also original, but consistent with the observations from the Helsinki Office Environment Study, which showed significantly increased risk of wheezing (OR = 1.29; 95% CI, 1.06–1.56) and chronic cough (OR = 1.43; 95% CI, 1.14–1.78) related to handling of self-copying paper (Jaakkola and Jaakkola 1999). LaMarte and coworkers (1988) from Iowa (USA) reported two cases who developed hoarseness, wheezing, and angioedema of arms when challenged with CCP. In one case this was accompanied by a 6-fold increase in plasma histamine levels and marked laryngeal edema after the challenge. The authors considered alkyphenol novolac resin as the agent responsible for these reactions.

We did not find any obvious relation between FPP exposure and asthma in office workers. Such a relation has not been addressed in previous studies. The Helsinki Office Environment Study did not find any significantly increased risk of chronic respiratory symptoms in relation to photocopying, although the point estimates for wheezing were somewhat increased (OR = 1.23; 95% CI, 0.99–1.53, for ≤ 4 hr/week exposure; OR = 1.26; 95% CI, 0.85–1.84, for > 4 hr/week exposure) (Jaakkola and Jaakkola 1999).

## Conclusions

This population-based study provides new evidence that exposures to paper dust and CCP in office work are related to increased risk of adult-onset asthma. A dose-dependent increase in the risk of asthma was observed in relation to increasing paper dust exposure. This exposure–response relation could be explained by an irritant mechanism, whereas the mechanism underlying CCP exposure and asthma could be sensitization that can take place even in small exposure levels. Reduction of these exposures could prevent asthma in office workers. The measures that can be introduced to reduce these exposures include reduced handling of the sources of exposure, improved ventilation where such handling is necessary, and use of alternative data storage and copying methods. Clinicians seeing asthma patients should be aware of this link to office work exposures.

## Figures and Tables

**Table 1:** Characteristics of the study population, The Finnish Environment and Asthma Study, 1997–2000.

Characteristic	Cases No. (%)	Controls No. (%)
Total no. of subjects	133	316
Sex		
Male	41 (30.8)	163 (51.6)
Female	92 (69.2)	153 (48.4)
Age (years)		
21–29	15 (11.3)	34 (10.8)
30–39	34 (25.6)	93 (29.4)
40–49	43 (32.3)	99 (31.3)
50–59	37 (27.8)	79 (25.0)
60–64	4 (3.0)	11 (3.5)
Education^a^		
No vocational schooling	13 (9.9)	21 (6.7)
Vocational course	18 (13.6)	20 (6.4)
Vocational institution	14 (10.6)	43 (13.7)
College-level education	53 (40.2)	134 (42.5)
University or corresponding	34 (25.8)	97 (30.8)
Smoking^b^		
No	66 (50.0)	178 (56.3)
Former	30 (22.7)	67 (21.2)
Current (regular or occasional)	36 (27.3)	71 (22.5)
ETS in the workplace or at home	16 (12.0)	37 (11.7)
Visible mold or mold odor in the workplace or at home	33 (24.8)	65 (20.6)

ETS, environmental tobacco smoke.

a Information on education was missing for 2 subjects.

b Information on smoking was missing for 1 subject.

**Table 2:** Distribution of exposures in cases (*n* = 133) and controls (*n* = 316), and crude and adjusted OR (95% CI) of asthma in relation to exposures to paper dust, CCP, and FPP, The Finnish Environment and Asthma Study, 1997–2000.

Exposure	Cases No. (%)	Controls No. (%)	Crude OR (95% CI)	Adjusted^a^ OR (95% CI)
Paper dust				
No exposure (reference)	47 (35.3)	178 (56.3)	1.00	1.00
Any exposure	86 (64.7)	138 (43.7)	2.36 (1.55–3.59)	1.97 (1.25–3.10)
Per 10 hr/week			1.24 (1.11–1.41)	1.20 (1.06–1.37)
1 to < 30 hr/week	28 (21.1)	58 (18.4)	1.83 (1.05–3.18)	1.47 (0.81–2.67)
30–60 hr/week	58 (43.6)	80 (25.3)	2.75 (1.72–4.38)	2.34 (1.41–3.89)
CCP				
No exposure (reference)	90 (67.7)	250 (79.1)	1.00	1.00
Any exposure	43 (32.3)	66 (20.9)	1.81 (1.15–2.85)	1.66 (1.03–2.66)
Per 10 hr/week			1.11 (0.95–1.30)	1.08 (0.92–1.27)
1 to < 15 hr/week	23 (17.3)	33 (10.4)	1.94 (1.08–3.47)	1.89 (1.02–3.49)
15–60 hr/week	20 (15.0)	33 (10.4)	1.68 (0.92–3.08)	1.45 (0.77–2.72)
FPP				
No exposure (reference)	82 (61.6)	206 (65.2)	1.00	1.00
Any exposure	51 (38.4)	110 (34.8)	1.17 (0.77–1.77)	1.06 (0.68–1.65)
Per 10 hr/week			1.16 (1.00–1.35)	1.12 (0.95–1.31)
1 to < 5 hr/week	17 (12.8)	47 (14.9)	0.91 (0.49–1.67)	0.90 (0.47–1.72)
5–60 hr/week	34 (25.6)	63 (19.9)	1.36 (0.83–2.21)	1.17 (0.69–1.95)
Any of the three office exposures^b^				
No exposure (reference)	38 (28.6)	145 (45.9)	1.00	1.00
Any of the three exposures	95 (71.4)	171 (54.1)	1.83 (1.04–3.22)	1.90 (1.05–3.44)

a Logistic regression analysis: adjusted for age, sex, education (as a measure of socioeconomic status), smoking status, exposure to ETS (at home and/or work), exposure to molds (at home and/or work).

b Any exposure to paper dust, CCP, and/or FPP.

## Appendix 1. Diagnostic criteria for asthma, The Finnish Environment and Asthma Study, 1997–2000

**Table:**

1. Occurrence of at least one asthmatic symptom: prolonged cough, wheezing, attacks of or exercise-induced dyspnea, or nocturnal cough or wheezing
*and*
2. Demonstration of reversibility in airways obstruction in lung function tests:
Significant improvement in response to short-acting bronchodilating medication in a bronchodilator test. The criteria for significant changes were:
FEV_1_ ≥ 15%
FVC ≥ 15%
PEF ≥ 23%
*and/or*
≥ 20% daily variation^a^ and/or ≥ 15% improvement^a^ in response to short-acting bronchodilating medication during at least 2 days in a 2-week diurnal PEF follow-up
*and/or*
Significant improvement in spirometric lung function (for % criteria see above) and/or ≥ 20% improvement in the average PEF level in response to a 2-week oral steroid treatment

FEV_1_, forced expiratory volume in 1 sec; FVC, forced vital capacity; PEF, peak expiratory flow.

a Calculated according to the standard practice of the Tampere University Hospital (National Asthma Program in Finland 1994): maximum daily variation = (highest PEF value during the day – lowest PEF value during the day)/highest PEF value during the day; bronchodilator response = (highest PEF value after bronchodilating medication – highest PEF value before medication)/highest PEF value before medication.
